# Cardioembolic Artery of Percheron Infarction

**DOI:** 10.7759/cureus.70515

**Published:** 2024-09-30

**Authors:** Lhara Monique L Llapitan, Bonifacio C Pedregosa, Jose C Navarro

**Affiliations:** 1 Department of Neurology, Jose R. Reyes Memorial Medical Center, Manila, PHL

**Keywords:** acute cerebral ischemic stroke, artery of percheron infarct, ischemic cerebrovascular disease, posterior circulation stroke, vascular neurology

## Abstract

Occlusion of the artery of Percheron (AOP) is a rare yet potentially disabling form of ischemic stroke resulting in infarction of the bilateral paramedian thalami and mesencephalon with variable and often atypical presentation. Given the various patterns of thalamic blood supply, recognizing the presence of AOP infarction is crucial for the diagnosis and management of ischemic strokes involving these regions. Here, we report a case of acute hemorrhagic infarction involving the bilateral thalami and the rostral mesencephalon caused by a cardioembolic occlusion of the AOP.

## Introduction

The artery of Percheron (AOP), first described by Gerard Percheron, is a rare variant of paramedian arterial supply wherein a single thalamoperforating artery originates from the proximal segment of the posterior cerebral artery and divides to supply both paramedian thalami and, in some instances, the rostral mesencephalon [1,2]. Occlusion of this artery thus results in a characteristic pattern of bilateral paramedian thalamic infarcts with or without involvement of mesencephalon [2,3].

## Case presentation

A 55-year-old male with no known comorbidities presented with a sudden onset decrease in sensorium. Upon examination, the patient was seen obtunded, with unsustained eye opening to pain, no verbal output, and an inability to follow commands. There was a forced downward deviation of both eyes, which did not correct with oculovestibular testing. Vital signs were within normal limits. The rest of his neurologic, cardiovascular, and general physical examination findings were unremarkable.

**Figure 1:**
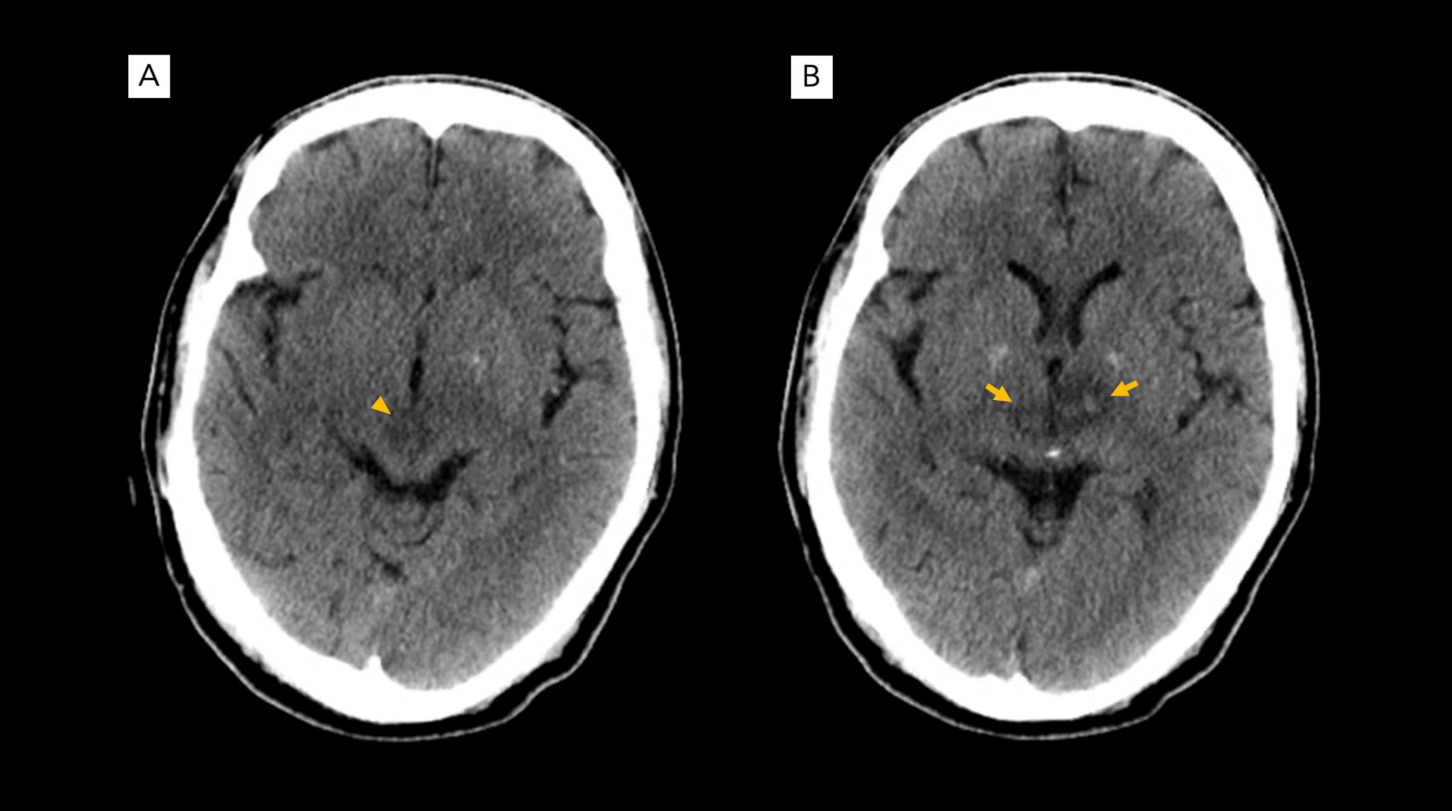
Cranial CT scan findings Non-contrast enhanced cranial CT scan taken eight hours post-ictus reveals an acute hemorrhagic infarction of the rostral mesencephalon (A, arrowhead) and bilateral thalami (B, arrows).

**Figure 2:**
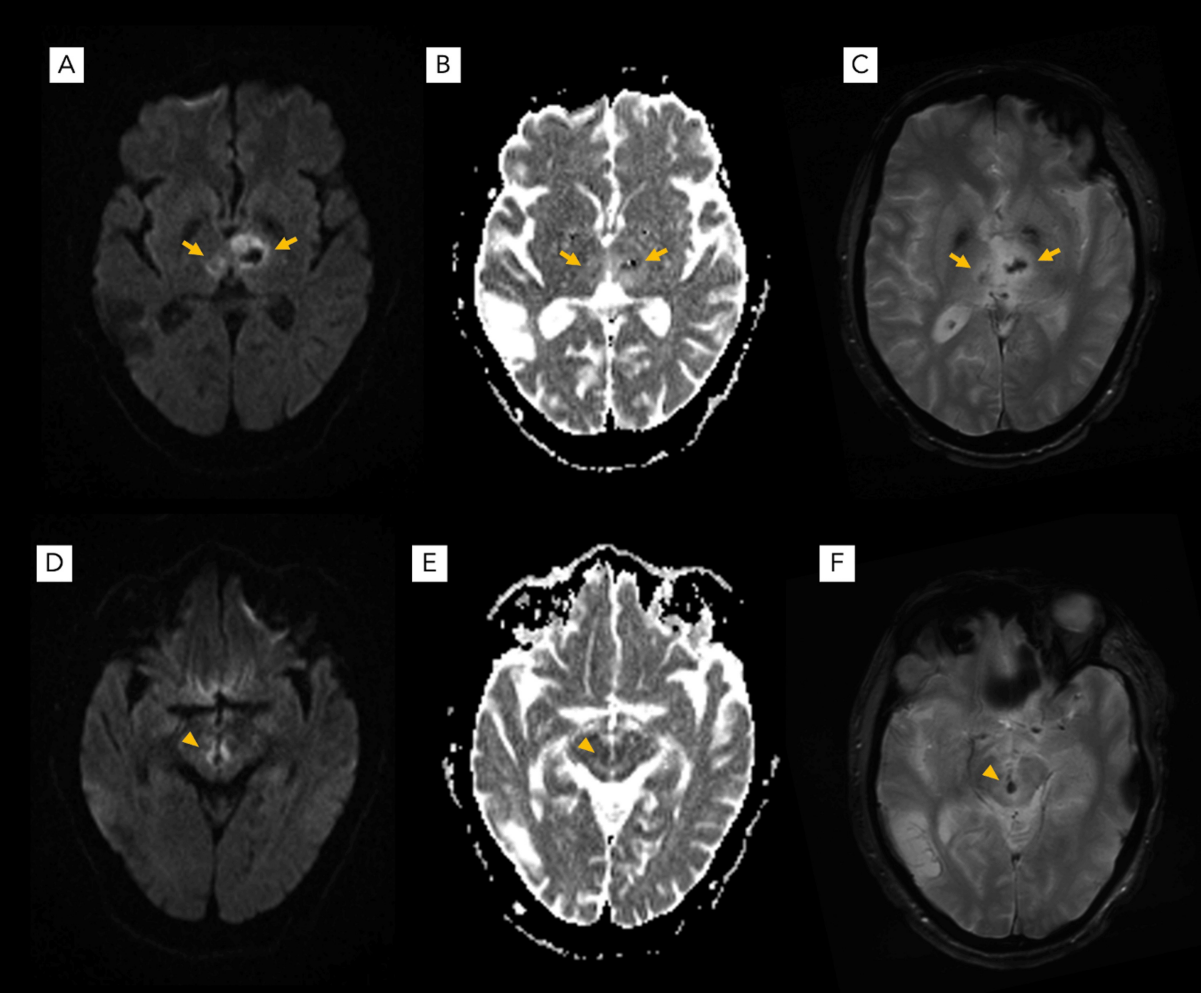
Cranial MRI findings Axial diffusion-weighted (A, D), apparent diffusion coefficient (B, E), and gradient recalled echo (C, F) MRI taken 10 hours post-ictus reveal an acute hemorrhagic infarction of the bilateral thalami (arrows) and rostral mesencephalon (arrowheads).

Initial electrocardiogram, biochemical laboratory tests, blood counts, and coagulation parameters were normal. The cranial CT scan (Figure 1) and subsequent cranial MRI (Figure 2) revealed an acute hemorrhagic infarction of the bilateral thalami and the rostral mesencephalon. An MR angiography was normal, demonstrating patency of the major arteries of the posterior circulation, including the basilar tip. The MR venography was unremarkable. Paroxysmal atrial fibrillation was noted on prolonged cardiac monitoring. Findings are consistent with an acute cardioembolic AOP infarction.

Our patient was eventually started on anticoagulation for secondary stroke prevention. The echocardiogram was normal with no noted valvular pathologies and an ejection fraction of 62%. He was discharged after two weeks of admission with no recurrent embolic events, and gradual resolution of hypersomnolence and aphasia. Forced downward deviation of both eyes persisted. 

## Discussion

Strokes affecting both thalamic nuclei in the context of patent basilar and posterior cerebral arteries should raise the suspicion of an AOP occlusion [1]. The AOP is a variant wherein the bilateral paramedian thalami are supplied by a single artery arising from the proximal segment of the posterior cerebral artery [2,3]. An AOP occlusion may lead to midbrain infarction through the superior mesencephalic (rubral) arteries [3]. Typical patterns of AOP infarction are as follows: bilateral paramedian thalamic infarctions with mesencephalic involvement (43%); bilateral paramedian thalamic infarctions without mesencephalic involvement (38%); and bilateral paramedian thalamic infarctions with involvement of the anterior thalamus and the mesencephalon (14%) [1].

Impairment in consciousness seen in AOP infarctions usually results from the disruption of the reticular activating system [4,5]. Whereas involvement of the rostral interstitial nucleus of the medial longitudinal fasciculus, the posterior commissure, the interstitial nucleus of Cajal, and the peri-aqueductal gray accounts for vertical gaze impairments [3-5]. 

The precise prevalence of AOP remains uncertain [6-8]. However, two autopsy studies have found AOP in 11.7% and 7% of the examined brains [6,7,9]. It has been reported that AOP occlusion accounts for 0.1% to 2.0% of all ischemic strokes [6,10], and 4% to 35% of all thalamic strokes [11]. Cardioembolism is the second most common etiology for AOP occlusion (33%), preceded by small vessel disease (40%) [1].

Fluid-attenuated inversion recovery (FLAIR) and diffusion-weighted imaging (DWI) are the preferred neuroimaging techniques for the early detection of AOP infarctions [3]. Due to its size, AOP identification through conventional angiography is difficult [3]. Management of AOP infarctions typically involves thrombolysis if the diagnosis is made within the established time window [12]. However, secondary stroke prevention varies depending on the pathophysiological etiology [12].

In a study assessing the long-term outcomes of AOP infarcts, prognosis was shown to be generally favorable, except in cases with mesencephalic involvement [11]. In the said study, favorable outcomes (defined by a modified Rankin scale (mRS) score of ≤2) were noted in 67% of patients who had bilateral paramedian thalamic infarctions without mesencephalic involvement. While only 25% of patients with both bilateral thalamic and rostral mesencephalic infarcts achieved similar outcomes.

## Conclusions

An AOP occlusion is an uncommon yet potentially disabling type of ischemic stroke that can present with a diverse range of symptoms depending on the distribution and extent of the infarct. Difficulty in detecting these lesions with cranial CT scans makes the diagnosis even more challenging. Timely recognition and management of AOP strokes are crucial for improving patient outcomes.
